# Gender discrimination against female surgeons: A cross-sectional study in a lower-middle-income country

**DOI:** 10.1016/j.amsu.2020.07.033

**Published:** 2020-07-24

**Authors:** Mahin B. Janjua, Hina Inam, Russell S. Martins, Nida Zahid, Abida K. Sattar, Shaista M. Khan, Sadaf Khan, Aneela Darbar, Nuzhat Faruqui, Sharmeen Akram, Syed A. Enam, Adil H. Haider, Mahim A. Malik

**Affiliations:** The Aga Khan University, Pakistan

## Abstract

**Introduction:**

Although gender discrimination and bias (GD/bias) experienced by female surgeons in the developed world has received much attention, GD/bias in lower-middle-income countries like Pakistan remains unexplored. Thus, our study explores how GD/bias is perceived and reported by surgeons in Pakistan.

**Method:**

A single-center cross-sectional anonymous online survey was sent to all surgeons practicing/training at a tertiary care hospital in Pakistan. The survey explored the frequency, source and impact of GD/bias among surgeons.

**Results:**

98/194 surgeons (52.4%) responded to the survey, of which 68.4% were males and 66.3% were trainees. Only 19.4% of women surgeons reported ‘significant’ frequency of GD/bias during residency. A higher percentage of women reported ‘insignificant’ frequency of GD/bias during residency, as compared to males (61.3% vs. 32.8%; p = 0.004). However, more women surgeons reported facing GD/bias in various aspects of their career/training, including differences in mentorship (80.6% vs. 26.9%; p < 0.005) and differences in operating room opportunities (77.4% vs. 32.8%; p < 0.005). The source was most frequently reported to be co-residents of the opposite gender. Additionally, a high percentage of female surgeons reported that their experience of GD/bias had had a significant negative impact on their career/training progression, respect/value in the surgical team, job satisfaction and selection of specialty.

**Conclusion:**

Although GD/bias has widespread impacts on the training/career of female surgeons in Pakistan, most females fail to recognize this GD/bias as “significant”. Our results highlight a worrying lack of recognition of GD/bias by female surgeons, representing a major barrier to gender equity in surgery in Pakistan and emphasizing the need for future research.

## Highlights

• Reported rates of overt gender discrimination remained low in our study.
• Respondents noted gender discrimination most significantly during their residency.
• Gender discrimination plays a significant role in mentoring and OR opportunities for women surgeons.

## 1 Introduction

Due to an increasing spotlight, gender discrimination and bias (GD/bias) is identified and reported in many aspects of surgical training and practice in the developed world [1,2]. While subjective gender discrimination remains hard to quantify, objective evidence of disparity is seen in the dearth of females in leadership positions, pay inequities, and underrepresentation in academics. Recent attention to GD in medicine and other walks of life has largely made overt bias widely unacceptable. However, women continue to face and deal with “unconscious” or implicit bias that results from an interplay of complex factors such as societal norms, stereotypical gender roles and learned behaviors [3].

In the last few years at least half of the enrolled medical students in the US are female [4], and while more women are choosing residencies in surgical specialties, there is still a significant absence at the top [5,6]. A 2017 study looking at women as department chairs and full professors showed dismal results, with few women making it beyond the rank of instructor and assistant professor, and even fewer making it to full professor and department chair [7]. Similar statistics are seen in other developed nations such as Great Britain and Japan, where Kyoto University noted no female associate or full professors in Surgery from 2009 to 2013 [8], and the Society of British Neurological Surgeons noted that out of 315 neurological surgeons, all 16 full professors were men [9].

In Pakistan, where ingrained cultural norms hinder women from practicing medicine even though they comprise 70% of the medical student population, it is highly likely that GD is quite prevalent in the field of surgery [10]. Thus, our study explores how GD is perceived and reported by surgeons practicing or training at a tertiary care hospital in Pakistan.

## 2 Methods

### 2.1 Study setting and population

This single-center cross-sectional study was carried out at an academic tertiary care hospital in Pakistan, from July–Sept 2019. Ethical approval for the study was obtained from the institutional review board. We used universal sampling whereby every person in the study population was approached. An anonymous online survey was sent to all consultants, fellows, instructors, and residents working in the Department of Surgery. The online survey was preceded by a consent form explaining the aim of the study, as well as the extent of the subject's involvement. A high degree of anonymity was maintained throughout the survey to minimize participation bias. The survey and its coding was adapted from a study by Bruce et al. [1], and was pilot tested prior to distribution to participants. It consisted of 100 units comprising the following sections:

### 2.2 Demographic and work characteristics

*Frequency of GD/Bias during Different Stages of Career*: A 10-Point Likert Scale was used to code frequency of GD/Bias as *None* 1(1), *Insignificant* (Scores [2], [3], [4]) and *Significant* (Scores [5], [6], [7], [8], [9], [10]].

### 2.3 Experience of GD/Bias in Different Aspects of career

*Sources of Experienced GD/Bias*: Options included Medical School Peers, Co-Residents, Nurses and Instructors.

### 2.4 *Impact of experienced GD/Bias on surgical career*

*Section for all Respondents*: A 5-Point Likert Scale was used to classify impact of GD/Bias as *None* [1(1), *Insignificant* (Scores 2–4) and *Significant* (Scores 5–10).

### 2.5 *Section for Attendings Only*: Responses were recorded as *Yes/No*

The STROCCS criteria was used and followed for reporting of this study [11].

### 2.6 Statistical analysis

Data was analyzed using SPSS (Statistical Package for Social Sciences) version 22.0. Qualitative variables were reported as frequencies and percentages. Furthermore, qualitative variables were compared using Chi-squared/Fisher Exact test. A p-value of <0.05 was considered significant throughout the study.

## 3 Results

A total of 194 surgeons were working or training at the tertiary care center at the time of the survey. Amongst these, 47 (24.2%) were female, with the highest representation amongst the surgical residents (34.4%) and lowest amongst the professors (9.1%) (Fig. 1a). The surgical specialties with the lowest representation of females were vascular surgery (none), orthopedic surgery (6.1%) and otolaryngology (11.1%). The highest representation of female surgeons was in breast surgery where 100% were females (Fig. 1b).

**Fig. 1a:**
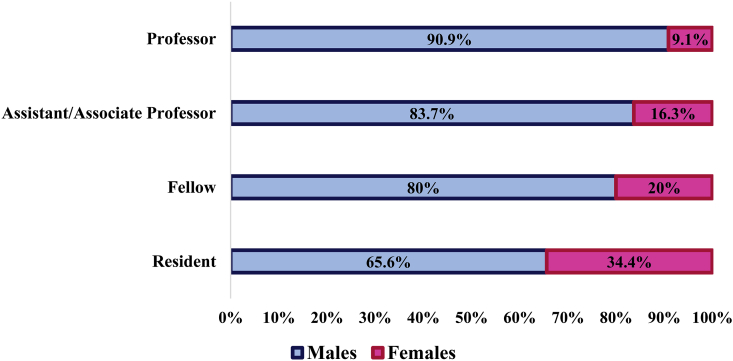
Gender distribution by position.

**Fig. 1b:**
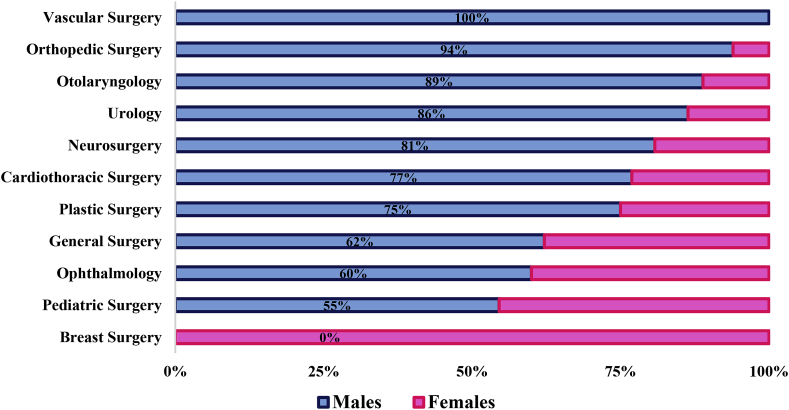
Gender distribution across specialty.

### 3.1 Demographics and work characteristics

A total of 98 surgeons (response rate 98/194 or 52.4%) were included in the study, out of whom 67 (68.4%) were male. The majority of respondents were trainees (n = 65, 66.3%) and the rest were practicing surgeons. Most respondents had attended a public sector medical school (61.2%). Ages were similar for males and females. More than half of both male and female surgeons worked for more than 80 h per week (Table 1). Respondents were most commonly from general surgery (n = 29; 29.6%), neurosurgery (n = 13; 13.3%) and orthopedic surgery (n = 12; 12.2%).

**Table 1:** Demographics, education and work characteristics of respondents.

Variable	Overall (N = 98) n (%)	Females (N = 31) n (%)	Males (N = 67) n (%)	P-Value
**Age**				
**25**–**35 Years**	67 (68.4)	23 (74.2)	44 (65.7)	
**35**–**45 Years**	12 (12.2)	4 (12.9)	8 (11.9)	
> **45 Years**	19 (19.4)	4 (12.9)	15 (22.4)	
**Current Position**				
**Trainee**	65 (66.3)	24 (77.4)	41 (61.2)	0.114
**Attending**	33 (33.7)	7 (22.6)	26 (38.8)	
**Medical School Sector**				
**Private**	38 (38.8)	13 (41.9)	25 (37.3)	0.662
**Public**	60 (61.2)	18 (58.1)	42 (62.7)	
**Work Hours/Week**				
**Part-time (** < **40 h)**	2 (2.0)	0 (0)	2 (3.0)	0.157
**40**–**80 h**	43 (43.9)	14 (45.2)	29 (43.3)	
**80**–**100 h**	29 (29.6)	13 (41.9)	16 (23.9)	
> **100 h**	24 (24.5)	4 (12.9)	20 (29.9)	

Although a higher percentage of women surgeons reported involvement in research (100% females vs. 80.6% males; p = 0.008), they reported significantly lower numbers of peer-reviewed publications than their male counterparts. Whilst the vast majority (87.1%) of female surgeons reported having 0–9 publications, 25.4% of male surgeons reported having 10–49 publications and 10.5% having > 50 publications (p = 0.036). None of the female surgeons in our sample reported having >50 publications.

### 3.2 Frequency of GD/Bias during Different Stages of Career

Only 19.4% of women reported *significant* frequency of GD/bias during residency. A significantly higher percentage of female surgeons reported “some’ frequency of GD/bias experienced during Residency (61.3% vs. 32.8%; p = 0.004). There was no significant difference between male and female respondents reporting GD/Bias experienced during medical school or surgical practice (Table 2).

**Table 2:** Frequency of Experience of GD/Bias during different Stages of Surgical Career.

	Females (N = 31) n (%)	Males (N = 67) n (%)	p-Value
**GD/Bias in Medical School**			
**Significant**	5 (16.1)	7 (10.4)	0.379
**Insignificant**	13 (41.9)	22 (32.8)	
**None**	13 (41.9)	38 (56.7)	
**GD/Bias in Residency**			
**Significant**	6 (19.4)	8 (11.9)	**0.004**
**Insignificant**	19 (61.3)	22 (32.8)	
**None**	6 (19.4)	37 (55.2)	
**GD/Bias in Practice (Attendings Only)**	**N = 7**	**N = 26**	
**Significant**	3 (42.9)	3 (11.5)	0.193
**Insignificant**	1 (14.3)	8 (30.8)	
**None**	3 (42.9)	15 (57.7)	

### 3.3 Experience of GD/Bias in Different Aspects of career

**Fig. 2:**
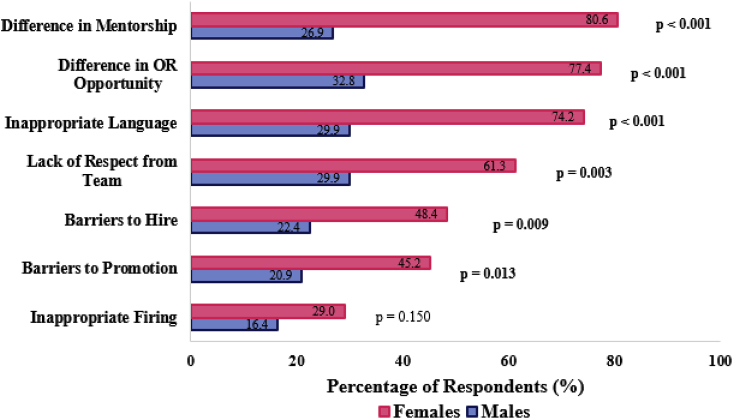
GD/bias in different aspects of carrer.

A significantly greater percentage of women surgeons reported having frequently experienced GD/Bias in various aspects of their surgical career. These included *differences in mentorship*, *differences in operating room (OR) opportunities*, *inappropriate language*, *lack of respect from team*, *barriers to hire* and *barriers to promotion* (Fig. 2).

### 3.4 Sources of Experienced GD/Bias

Amongst those reporting GD/bias experienced at any point in their surgical career (n = 61; 62.2%), the source of GD/bias was reported to be predominantly the opposite sex. Female respondents reported the source of GD/bias as males 60% of the time, and male respondents reported similarly for female sources (57.8%). For female respondents, the most common male source was *male co-residents*, and for male respondents the most common female source was *female co-residents* (Table 3). Amongst a total of 175 sources of GD/bias reported, the most common overall source of GD/bias irrespective of gender was *co-residents* (33.1%), followed by *medical school peers* (23.4%).

**Table 3:** Sources of experienced GD/Bias.

Source of Experienced GD/Bias	Female Respondents (N = 27)		Male Respondents (N = 34)	
	Source: Male	Source: Female	Source: Male	Source: Female
	**N (%)**	**N (%)**	**N (%)**	**N (%)**
**Medical School Peers**	11 (21.6)	7 (20.6)	11 (28.9)	12 (23.1)
**Co-Residents**	16 (31.4)	12 (35.3)	11 (28.9)	19 (36.5)
**Instructors**	13 (25.5)	5 (14.7)	8 (21.1)	10 (19.2)
**Nurses/OR Staff**	11 (21.6)	10 (29.4)	8 (21.1)	11 (21.2)
**TOTAL INSTANCES OF EXPERIENCES OF GD/Bias**	51	34	38	52
**% OF TOTAL INSTANCES OF EXPERIENCES OF GD/Bias**	60%	40%	42.2%	57.8%

### 3.5 Impact of experienced GD/Bias on surgical career

A significantly higher percentage of female surgeons reported that their experience of GD/bias had had a *significant* negative impact on their *career/training progression*, *respect/value in the surgical team*, *job satisfaction* and *selection of specialty*. Amongst surgeons in practice, a significantly higher percentage of female surgeons reported that their experience of GD/bias had negatively impacted their *access to leadership positions*, *career opportunities* and *referrals from other doctors* (Table 4). However, there were was no impact on quality of patient care for either groups.

**Table 4:** Negative impact of experienced GD/Bias on different aspects of career.

Aspect of Career * (All Respondents)	Females (N = 31) n (%)	Males (N = 67) n (%)	p-Value
**Career/Training Progression**			
**Significant**	25 (80.6)	22 (32.8)	< **0.001**
**Insignificant**	4 (12.9)	18 (26.9)	
**Respect/Value in Surgical Team**			
**Significant**	22 (71.0)	23 (34.3)	**0.001**
**Insignificant**	7 (22.6)	18 (26.9)	
**Job Satisfaction**			
**Significant**	21 (67.7)	21 (31.3)	**0.001**
**Insignificant**	7 (22.6)	20 (29.9)	
**Quality of Patient Care**			
**Significant**	13 (41.9)	20 (29.9)	0.261
**Insignificant**	11 (35.5)	21 (31.3)	
**Selection of Specialty**			
**Significant**	20 (64.5)	24 (35.8)	**0.028**
**Insignificant**	6 (19.4)	21 (31.3)	
Aspects of Career ** (Attendings Only)	**Females (N** = **7) n (%)**	**Males (N** = **26) n (%)**	**p-Value**
**Access to Leadership Positions**	3 (42.9)	0 (0)	**0.006**
**Career Opportunities**	5 (71.4)	3 (11.5)	**0.004**
**Referrals from Other Doctors**	7 (100)	11 (42.3)	**0.009**

* Responses coded “*None*” are not shown in section for All Respondents.

** Responses coded “*No*” are not show in section for Attendings Only.

**Fig. 3:**
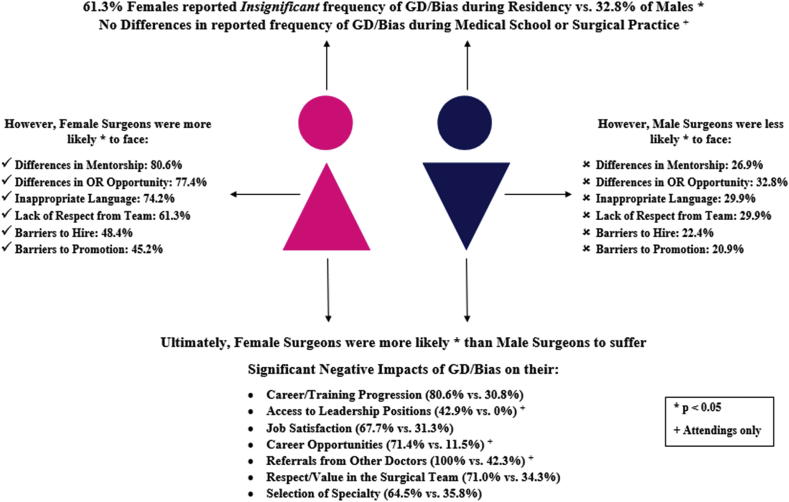
Summary of results.

Fig. 3 summarizes the results of our study.

## 4 Discussion

In this study, we aimed to examine the incidence and the overall experience of gender discrimination and bias (GD/bias) experienced by practicing surgeons and surgeons in-training at our institution. Our study shows that only 19.4% of female surgeons reported significant GD during residency, while 42.9% experienced GD during surgical practice. In comparison, a study by Bruce et al. conducted in the United States showed that 53%, 67%, and 68% of respondents experienced *significant* gender discrimination during medical school, residency and practice, respectively. However, only 19% of female surgery residents reported having experienced *no* gender discrimination during residency, which is similar to the findings in our study [1,2].

In the light of recent social movements (#MeToo, #TimesUp, #ILookLikeASurgeon) [11], women in traditionally male-dominated professions have made great strides in reporting and calling out GD/bias and harassment. However, reporting and understanding more insidious discriminatory behaviors remains under-recognized and largely unaddressed. Devine et al. describes implicit bias as ‘deeply entrenched habits developed through socialization experiences’ [12], essentially a learned behavior that is a result of societal and institutional culture. Like their counterparts in other professions, female surgeons have to toe a thin line around these gender stereotypes. Female doctors are frequently presumed to be nurses and are often treated with less respect [13]. This was mirrored in our study where disproportionately high percentages of women surgeons reported having faced significant problems in several key aspects of their career due to their gender. These included differences in mentorship and OR opportunities, experience of inappropriate language, and a lack of respect from the surgical team.

Since only 19.4% of women surgeons reported having faced significant GD/bias during residency, these findings indicate a worrying lack of recognition of GD/bias rather than a lack of experience. Rather than perceiving these as instances of GD/bias, these women may in fact be learning to accept that ‘this is just how things are’. Indeed, a longitudinal analysis of female medical students at a New England medical school showed that female students become acculturated to inappropriate experiences, ultimately resigning themselves to the inevitability of negative gendered experiences in their clinical training [14]. In a Muslim majority country, these gender roles and stereotypes are even more amplified due to certain cultural practices. These range from stereotypical gender-role perceptions to widespread societal dissuasion of females aspiring to pursue surgical careers [10,[15], [16], [17], [18], [19]]. This constant experience of normative gender bias may desensitize females to recognizing overt GD/bias [3]. In Pakistan, the limited importance given to addressing GD/bias in surgery could lead to a lack of awareness regarding the realistic achievability of better gender equity. This could contribute to the poor recognition of GD by women, despite its overarching experience in many aspects of their surgical career, as seen in our study.

Though more women are joining surgical residencies and are on track to represent half of all general surgery trainees by 2026, when analyzed for higher levels of academic leadership, it is projected that it will take another 100 years (2096–2136) till parity is reached at the full professor level [20]. Our data is just as alarming, where although 34.4% of residents are female, there was only one female full professor in the Department of Surgery. A study by Cochran at el. reports that women in academic surgery were 10 times more likely to have experienced gender discrimination than men [21]. Several studies have investigated the reasons behind gender disparities in surgery, as well as ways to lessen this gap [[21], [22], [23], [24], [25]]. The reasons attributed most commonly are: lack of role models and mentors, gender discrimination and lifestyle concerns. This undoubtedly plays a role in attrition, job satisfaction, likelihood for promotion, and ability to positively persuade female medical students to pursue a career in surgery.

Advancement in academic medicine is a complex, multi-layered process, with research productivity and publications being a critical and quantifiable factor. Several studies have shown that men have a higher number of publications and research indices (such has h-index, m-quotient) than women [[26], [27], [28]]. One hypothesis is that women are less productive than men due to early career demands alongside familial responsibilities [29]. Whilestudy shows similar results, with women surgeons having a lower total number of publications, working hours are similar. Whilst 87.1% of female surgeons reported having only 0–9 publications, 25.4% of males reported having 10–49 publications and 10.5% having >50 publications. However, further investigations are needed to better understand the effect of position and years of practice on these results.

Our study was limited by the small sample size, and its data being from a single center in Pakistan which could hinder the generalizability of our findings to the rest of the country. Additionally, GD/bias originating from patients was not explored in this survey, providing a potential for further cross-sectional research in this regard. However, the high degree of anonymity maintained throughout the survey and study helped minimize participation bias. This study is the first of its kind done in a Muslim majority lower-middle-income country and highlights the non-recognition of GD/bias as a major challenge to achieving gender equity in this setting. Recent efforts by women surgeon leaders in Pakistan include establishing the Association of Women Surgeons of Pakistan, an organization that aims to ‘aspire and mentor the current and next generation of women surgeons’, with the support of our institution. Such efforts need to be encouraged and supported at other institutions as well. Committees within surgical societies in Pakistan, such as Women in Neurosurgery and Women in Ophthalmology have already been established through the efforts of women surgeon leaders, giving women surgeons a united voice and a platform. Further work and efforts are needed to counter these biases and allow women surgeons to progress at par with their male colleagues.

## 5 Conclusion

Gender discrimination and bias (GD/bias) adversely impacts several key aspects of the careers of female surgeons in Pakistan, including career/training opportunities, access to leadership positions, respect/value in the surgical team and job satisfaction. Thus, our study highlights a worrying lack of recognition of GD/bias by female surgeons, representing a major barrier to gender equity in surgery in Pakistan. Women continue to be under-represented at the top of the hierarchy and additional research is warranted to better understand the barriers to progression of careers of women surgeons.

## Provenance and peer review

Not commissioned, externally peer reviewed.

## Author contribution

Mahin B Janjua: Data collection, data analysis, writing

Hina Inam: Study design, data collection, writing

Russell S Martins: Data analysis, writing

Nida Zahid: Data Analysis

Syed A Enam: Study design, writing

Adil H Haider: Study design

Mahim A Malik: Study design, data collection, data analysis, writing

## Please state any conflicts of interest

None

## Please state any sources of funding for your research

None

## Please state whether ethical approval was given, by whom and the relevant Judgement's reference number

Approved by Institutional Ethical Review Committee.

ERC number: 2019-1603-4171.

## Research registration Unique Identifying number (UIN)

1. Name of the registry: ClinicalTrials.gov Protocol Registration and Results System.

2. Unique Identifying number or registration ID: NCT04395313.

3. Hyperlink to your specific registration (must be publicly accessible and will be checked): https://clinicaltrials.gov/ct2/show/NCT04395313.

## Guarantor

Mahim A Malik.

## Funding

This study was not funded by any funding or grant agency.

## Declaration of competing interest

None

## References

[1] Bruce A.N., Battista A., Plankey M.W., Johnson L.B., Marshall M.B. (2015). Perceptions of gender-based discrimination during surgical training and practice. Med. Educ. Online.

[2] Hu Y.-Y., Ellis R.J., Hewitt D.B., Yang A.D., Cheung E.O., Moskowitz J.T. (2019). Discrimination, abuse, harassment, and burnout in surgical residency training. N. Engl. J. Med..

[3] Phillips N.A., Tannan S.C., Kalliainen L.K. (2016). Understanding and overcoming implicit gender bias in plastic surgery. Plast. Reconstr. Surg..

[4] Colleges AoAM. (2018). Fall Applicant and Matriculant Data Tables. 2018.

[5] Jonasson O. (2002). Leaders in American surgery: where are the women?. Surgery.

[6] Weiss A., Lee K.C., Tapia V., Chang D., Freischlag J., Blair S.L. (2014). Equity in surgical leadership for women: more work to do. Am. J. Surg..

[7] Epstein N.E. (2017). Discrimination against female surgeons is still alive: where are the full professorships and chairs of departments?. Surg. Neurol. Int..

[8] Okoshi K., Nomura K., Fukami K., Tomizawa Y., Kobayashi K., Kinoshita K. (2014). Gender inequality in career advancement for females in Japanese academic surgery. Tohoku J. Exp. Med..

[9] Wilkes F.A., Akram H., Hyam J.A., Kitchen N.D., Hariz M.I., Zrinzo L. (2015). Publication productivity of neurosurgeons in great Britain and Ireland. J. Neurosurg..

[10] Moazam F., Shekhani S. (2018). Why women go to medical college but fail to practise medicine: perspectives from the Islamic Republic of Pakistan. Med. Educ..

[11] Choo E., Byington C., Johnson N.-L., Jagsi R. (2019). From #MeToo to #TimesUp in health care: can a culture of accountability end inequity and harassment?. Lancet.

[12] Devine P.G., Forscher P.S., Austin A.J., Cox W.T.L. (2012). Long-term reduction in implicit race bias: a prejudice habit-breaking intervention. J. Exp. Soc. Psychol..

[13] Houry D., Shockley L.W., Markovchick V. (2000). Wellness issues and the emergency medicine resident. Ann. Emerg. Med..

[14] Babaria P., Abedin S., Berg D., Nunez-Smith M. (1982). I'm too used to it": a longitudinal qualitative study of third year female medical students' experiences of gendered encounters in medical education. Soc. Sci. Med..

[15] Inam H., Janjua M., Martins R.S., Zahid N., Khan S., Sattar A.K. (2020). Cultural barriers for women in surgery: how thick is the glass ceiling? An analysis from a low middle-income country. World J. Surg..

[16] Numann P. (2020). Cultural barriers for women in surgery: how thick is the glass ceiling? An analysis from a low middle-income country. World J. Surg..

[17] Inam H., Janjua M., Malik M. (2020). Authors' reply: cultural barriers for women in surgery: how thick is the glass ceiling? An analysis from a low middle-income country. World J. Surg..

[18] Ma X., Luc J. (2020). Letter to the editor: cultural barriers for women in surgery—how thick is the glass ceiling? An analysis from a low-middle-income country. World J. Surg..

[19] Kim E., Velin L., Mazhiqi A., Wall K., Vervoort D., Anderson E. (2020). Letter to the editor: cultural barriers for women in surgery: how thick is the glass ceiling? An analysis from a low-/middle-income country. World J. Surg..

[20] Sexton K.W., Hocking K.M., Wise E., Osgood M.J., Cheung-Flynn J., Komalavilas P. (2012). Women in academic surgery: the pipeline is busted. J. Surg. Educ..

[21] Cochran A., Hauschild T., Elder W.B., Neumayer L.A., Brasel K.J., Crandall M.L. (2013). Perceived gender-based barriers to careers in academic surgery. Am. J. Surg..

[22] Colletti L.M., Mulholland M.W., Sonnad S.S. (1960). Perceived obstacles to career success for women in academic surgery. Arch. Surg. (Chicago, Ill.

[23] Longo P., Straehley C.J. (2008). Whack! I've hit the glass ceiling! Women's efforts to gain status in surgery. Gend. Med..

[24] Neumayer L., Kaiser S., Anderson K., Barney L., Curet M., Jacobs D. (2002). Perceptions of women medical students and their influence on career choice. Am. J. Surg..

[25] Schroen A.T., Brownstein M.R., Sheldon G.F. (2004). Women in academic general surgery. Academic medicine. J. Assoc. Am. Med. Colleges.

[26] Lopez J., Susarla S.M., Swanson E.W., Calotta N., Lifchez S.D. (2015). The association of the H-index and academic rank among full-time academic hand surgeons affiliated with fellowship programs. J. Hand Surg..

[27] Diamond S.J., Thomas C.R., Desai S., Holliday E.B., Jagsi R., Schmitt C. (2016). Gender differences in publication productivity, academic rank, and career duration among U.S. Academic gastroenterology faculty. Academic medicine. J. Assoc. Am. Med. Colleges.

[28] Mueller C.M., Gaudilliere D.K., Kin C., Menorca R., Girod S. (2016). Gender disparities in scholarly productivity of US academic surgeons. J. Surg. Res..

[29] Zhuge Y., Kaufman J., Simeone D.M., Chen H., Velazquez O.C. (2011). Is there still a glass ceiling for women in academic surgery?. Ann. Surg..

